# Modeling Occupancy of Hosts by Mistletoe Seeds after Accounting for Imperfect Detectability

**DOI:** 10.1371/journal.pone.0127004

**Published:** 2015-05-14

**Authors:** Rodrigo F. Fadini, Renato Cintra

**Affiliations:** 1 Programa de Pós-Graduação em Ecologia, Instituto Nacional de Pesquisas da Amazônia, CP 478, Manaus, 69067–375, AM, Brazil; 2 Coordenação de Biodiversidade, Instituto Nacional de Pesquisas da Amazônia, Manaus, 69067–375, AM, Brazil; University of Vigo, SPAIN

## Abstract

The detection of an organism in a given site is widely used as a state variable in many metapopulation and epidemiological studies. However, failure to detect the species does not necessarily mean that it is absent. Assessing detectability is important for occupancy (presence—absence) surveys; and identifying the factors reducing detectability may help improve survey precision and efficiency. A method was used to estimate the occupancy status of host trees colonized by mistletoe seeds of *Psittacanthus plagiophyllus* as a function of host covariates: host size and presence of mistletoe infections on the same or on the nearest neighboring host (the cashew tree *Anacardium occidentale*). The technique also evaluated the effect of taking detectability into account for estimating host occupancy by mistletoe seeds. Individual host trees were surveyed for presence of mistletoe seeds with the aid of two or three observers to estimate detectability and occupancy. Detectability was, on average, 17% higher in focal-host trees with infected neighbors, while decreased about 23 to 50% from smallest to largest hosts. The presence of mistletoe plants in the sample tree had negligible effect on detectability. Failure to detect hosts as occupied decreased occupancy by 2.5% on average, with maximum of 10% for large and isolated hosts. The method presented in this study has potential for use with metapopulation studies of mistletoes, especially those focusing on the seed stage, but also as improvement of accuracy in occupancy models estimates often used for metapopulation dynamics of tree-dwelling plants in general.

## Introduction

A metapopulation is a group of local populations patchily distributed where migration is possible at least between some populations [1]. One of the models most used for understanding metapopulation dynamics is the “incidence function model” (IFM) [2–4], in which the processes of colonization and extinction are inferred from spatially explicit data collected on the occurrence patterns of organisms in their habitat patches on successive sampling [5]. In this model, probabilities of colonization and extinction, which are independent for every patch in each time period [3,6] are modelled as a function of the degree of patch isolation and size, respectively, where colonization is higher for well-connected patches, and extinction is lower for larger patches [2,3].

Many key applications of metapopulation models were first made in the field of epidemiology [7,8]; hosts are analogous to habitat patches that can be occupied by organisms (parasites). As in metapopulations, in host-parasite models the characteristics of the focal host and of its neighborhood are more important for determining the probability of its infection than for the whole host population [9], making the model of parasite transmission similar to the IFM in such scenarios.

Mistletoes, which are aerial hemiparasitic plants of the Sandalwood order (Santalales), are excellent model organisms for studying metapopulation dynamics and disease transmission because of the facilities they offer for testing theoretical predictions through manipulation and field observation [10–14]. Hosts are habitat patches connected by seed dispersal (migration). Colonization occurs when the seed adheres and establishes on the host tree, while extinction occurs through the death of the infecting plants or of the host itself. Many mistletoe species are dispersed by birds (seed vectors), which remove the fruits from infected trees and deposit the majority of seeds on tree branches, especially in well-connected, large hosts [15–18], a pattern similar to that proposed by the IFM.

In pursuit of these model organisms, it is necessary to investigate and quantify a problem that has been identified for more than a decade by several researchers studying metapopulations—that of false absences—that is, the failure to detect a given species in a habitat patch when it is, in fact, present [19–22]. This problem may occur because the species is inconspicuous [23,24], or because its abundance is so low that it negatively influences its detection [25,26]. It is important to take such problems into account because, if species detection is low, it can lead to underestimates of colonization, resulting in, among others, underestimations of dispersal distance, and overestimation of extinction rates [27,28].

To take detectability into account, Mackenzie et al. [20,21] proposed a model to estimate the incidence of a target species using information gathered from repeated-surveys (hereafter re-survey) conducted at the same site or patch. In this method, occupancy (Ψ) (the probability of a species being present at a site in a given survey) and detectability (*p*) (the probability of species detection at a site given its presence) can be modeled simultaneously using a technique that allows the inclusion of covariates influencing both parameters.

We used re-surveys of mistletoe seeds on host trees to evaluate the issue of false-absences and quantify its influence on occupancy estimates. In fact, before conducting this study, we had already noted detection failures when we misclassified some hosts as occupied by seeds when, indeed, they were not (RFF, unpublished data). Although recording the detection/non-detection of mistletoes at sampling sites (i.e., hosts) is less time-consuming than collecting detailed data on absolute or relative abundance within host tree canopies, detection of seeds or of established mistletoe plants on host trees is not always an easy task. The large size of some host trees, visual obstruction of parts of host canopy, low abundance of mistletoe seeds within host crowns, plus the rarity and low conspicuity of some species, may all reduce their detectability.

Here we used the mistletoe *Psittacanthus plagiophyllus* Eichl. (Loranthaceae) as a model study organism because, at our study site, it is locally specialized on the cashew tree *Anacardium occidentale* L. (Anacardiaceae) [29], which eliminates the influence of different host species affecting detection probabilities. We evaluated three predictions relating to detectability and to occupancy: (1) detectability of mistletoe seeds is negatively affected by host size, due to the difficulty of locating mistletoe seeds on larger hosts. For example, it could be easier to detect at least one mistletoe seed in a small host tree, than in a bigger tree with the same number of seeds. In contrast, occupancy increase with host size because of the preference of birds to perch and deposit seeds on larger than average hosts [30,31]; (2) detectability of mistletoe seeds is positively affected by proximity to infected neighbors, the same occurs for occupancy. This is because hosts with infected neighbors have a higher probability of receiving mistletoe seeds than isolated ones, increasing detection and occupancy probabilities; (3) previously infected hosts attract bird seed dispersers more often than non-infected hosts, which, in turn, deposit seeds frequently on such trees, increasing occupancy. Consequently, detectability of mistletoe seeds should also be higher on previously parasitized hosts simply because they have more seeds. The study assumed these mechanisms were operating, but they were not investigated directly.

Finally, we hypothesized that not accounting for detectability would underestimate the occupancy estimates of host trees. To evaluate this, we compared the naïve occupancy estimates obtained using a logistic model fitted to our data, with the occupancy estimates of the best model we fitted after accounting for detectability. We conclude discussing the issue of detectability not only for theoretical metapopulation mistletoe studies, but also for monitoring programs of established mistletoes and other tree-dwelling plants conducted at larger spatial scales.

## Materials and Methods

### Study site and species

**Fig 1:**
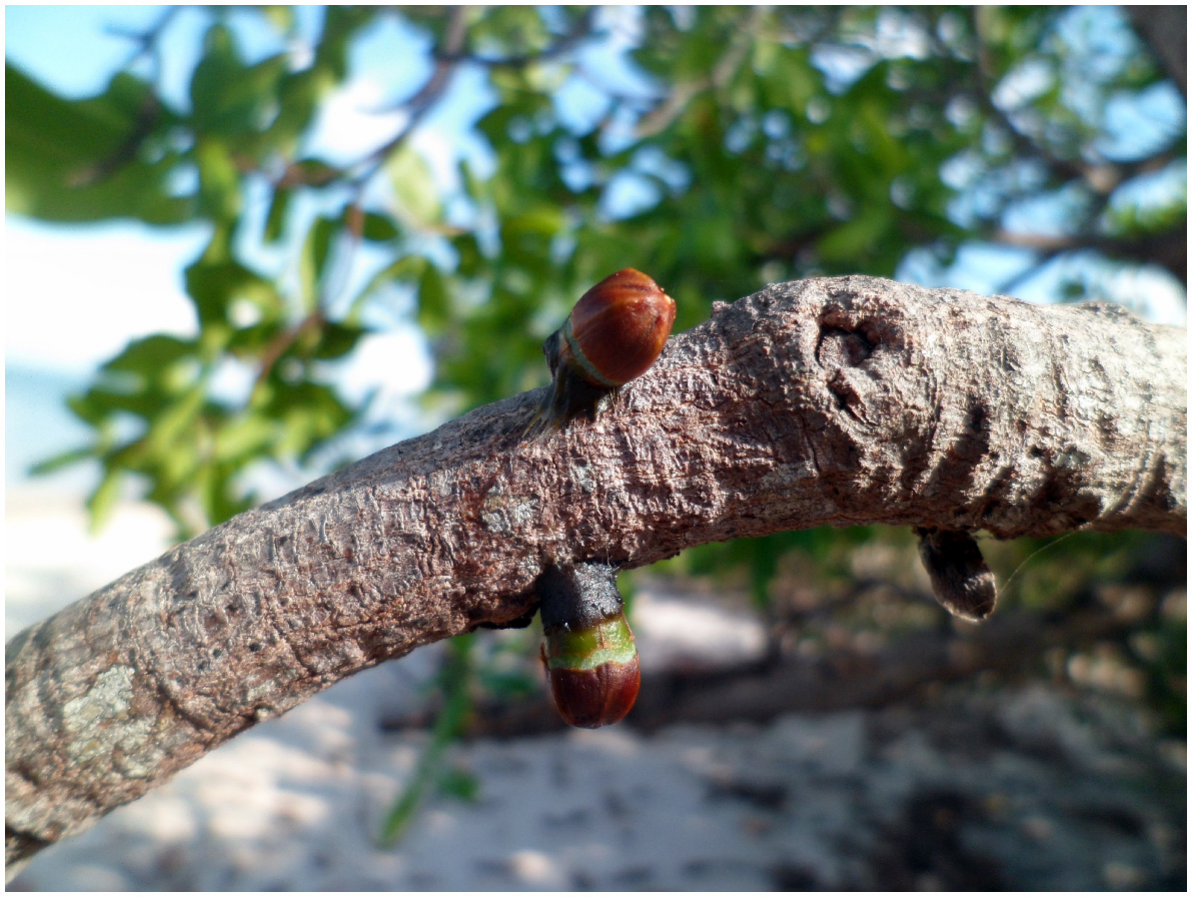
Seeds of *P*. *plagiophyllus* attached to a branch of *A*. *occidentale* (Photo: Leidielly Ghizoni).

The study was conducted in a large (10 x 10 km) patch of savanna on the right bank of the Tapajós River, near Alter do Chão (2°31′00” S, 54°57′02” W), Santarém, Pará, Brazil. Our study species, *Psittacanthus plagiophyllus*, is a shrubby hemiparasite that occurs in savannas of northern South America [32]. It has large, yellow/orange, hummingbird pollinated flowers, and black, elliptical fruits (length = 11.25 mm ± 0.56, width = 8.86 mm ± 0.38, N = 80). Fruits are dispersed from mid-June to late September mainly by the Plain-crested Elaenia (*Elaenia cristata*) [17], a tyrant flycatcher common in Brazilian savannas and cerrados, which ingests the fruits whole and drops seeds by regurgitation or bill-wiping on tree branches [33]. Seeds are small (length = 10 mm ± 0.7, width = 7.8 mm ± 0.7, N = 100) (Fig 1). Established adult mistletoes can be large (74 ± 13.4 cm of diameter, N = 11) and are frequently aggregated within host trees.

### Repeated detection / non-detection surveys

Twenty-four to 28 cashew trees (total of 130 hosts) were randomly selected in each of five host populations, and three simultaneous surveys were conducted on the same host trees between 5 and 24 August 2008, when the majority of mistletoe seeds had been dispersed. We chose study individuals from the cashew tree hosts using a random number table to provide angles between 0 and 259 degrees. We walked 50 m in the direction drawn, and then marked the nearest host tree with an aluminum tag. All host trees included in the analysis were distant from each other by at least 50 m: if a drawn point indicated a host shorter than this minimum distance, then a new point was drawn. All hosts included in the study were georeferenced with a hand-held GPS. For three populations (80 hosts), two observers, one at a time, climbed host trees and searched independently for mistletoe seeds. There was no communication between the observers during and after the searching period, which lasted 3 min (the same sampling effort used for more than 90% of time-free surveys of mistletoe seeds in this host species, R. Fadini unpublished data). We used a fixed time because the observers could influence one another if they had a free searching time, in the sense they have a tendency of leaving the tree soon after all mistletoe seeds are found. Both observers recorded detection or non-detection of mistletoe seeds on a spreadsheet immediately following the survey. For the remaining two host populations (50 hosts), there were three observers instead of two. Only RF sampled all populations.

We chose three site (host) covariates to account for variation in mistletoe detectability and occupancy: (1) “host size” (crown diameter in meters); (2) presence/absence of infected hosts with fruit-producing mistletoes within 50 m of focal hosts (hereafter “presence of infected neighbor”); (3) presence/absence of adult, fruit producing mistletoes on the focal host (hereafter “presence of infection”). Furthermore, because host size is frequently correlated with the abundance of mistletoe seeds [15, but see 34], more seeds would be “diluted” in a bigger tree and, therefore, host size “controls” the effect of seed abundance, being a good covariate for detectability.

### Statistical analyses

Each host tree that was searched for mistletoe seeds had its own detection/non-detection history composed of a sequence of ones (detection) and zeros (non-detection), corresponding to observations of the same host made by multiple observers for three-minute periods. First, the probability of a host being occupied or not is described, respectively, as Ψ and (1-Ψ). If the host is unoccupied, mistletoe seeds were not detected there. If the site is occupied, the mistletoe seed can be detected with probability *p* or not detected with probability 1-*p*. For example, the detection history hi = 010 is described as: the species is present in this host (at least one “1” in the history). Therefore, it is present but not detected on the first occasion, detected on the second, and present but non-detected on the third. The probability of observing this detection history can be described as:

Pr(hi=010)=ψ(1−p)p(1−p)

In an extreme case, a given host may have the history Pr (hi = 000). Therefore, the mistletoe seed may not be present at this host *or* it may be present but go undetected in the three surveys. This can be described as:

$$\mathrm{Pr}(hi=000)=\psi \prod_{j=1}^{3}\left(1-p)+(1-\psi \right)$$

All these models assume that probabilities are constant across all hosts [represented by *p*(.)]. However, probabilities of detection may vary according to host characteristics (covariates) such as host size [*p*(host size)], proximity to infected hosts [*p*(neighbor)], and presence of adult infections [*p*(infection)] [35]. We can model this using the *logit* link function [36], expressing the probability of a host tree being detected with a mistletoe seed according to β host covariates, where the subscripts “i” and “j” denotes the individual host and the survey occasion, respectively.

Logit(piji)=β0+β1xi1+…+βUxiU

Similarly, we can model the probability of a host tree being occupied according to the same or different host covariates (as for detection probabilities):

Logit(ψ)=β0+β1xi1+…+βUxiU

The product of all detection/non-detection histories at all hosts generated a likelihood model which is maximized to obtain maximum likelihood estimates of *p* (detectability) (detectability term) and Ψ (occupancy) (occupancy term). We constructed thirty-six models performing a full combination of covariates in the occupancy and detectability, except those containing both “host size” and “presence of infection” in the same term because of multicolinearity problems. We pooled all sites for analysis because we wanted to model occupancy and detectability for focal hosts rather than for study sites. This is in accordance with IFM [2] as well as with more spatially explicit epidemiological models [9], where the state of a given patch or host is modeled as a function of their own characteristics and neighborhoods. Comparison among models was made using a parsimonious penalized likelihood function ranked by the AIC [37] provided by the software Presence 2.0 [38]. Models with AIC differences less than 2 have substantial support, 4 to 7 have less support, and greater than 10 have no support. The ordering criteria of models is based on the relative AIC corrected for small samples (ΔAICc). The model averaging (W) is used when the best models are not separated by a difference of AIC larger than 2. Raw results were expressed as means with standard deviations, except on graphs that represented means with confidence intervals.

### Ethics statement and public repository data

No specific permissions were required for this study. Field work did not involve endangered or protected species.

## Results

The data underlying the statistical analyses in the present study can be found in (S1 Dataset).

**Fig 2:**
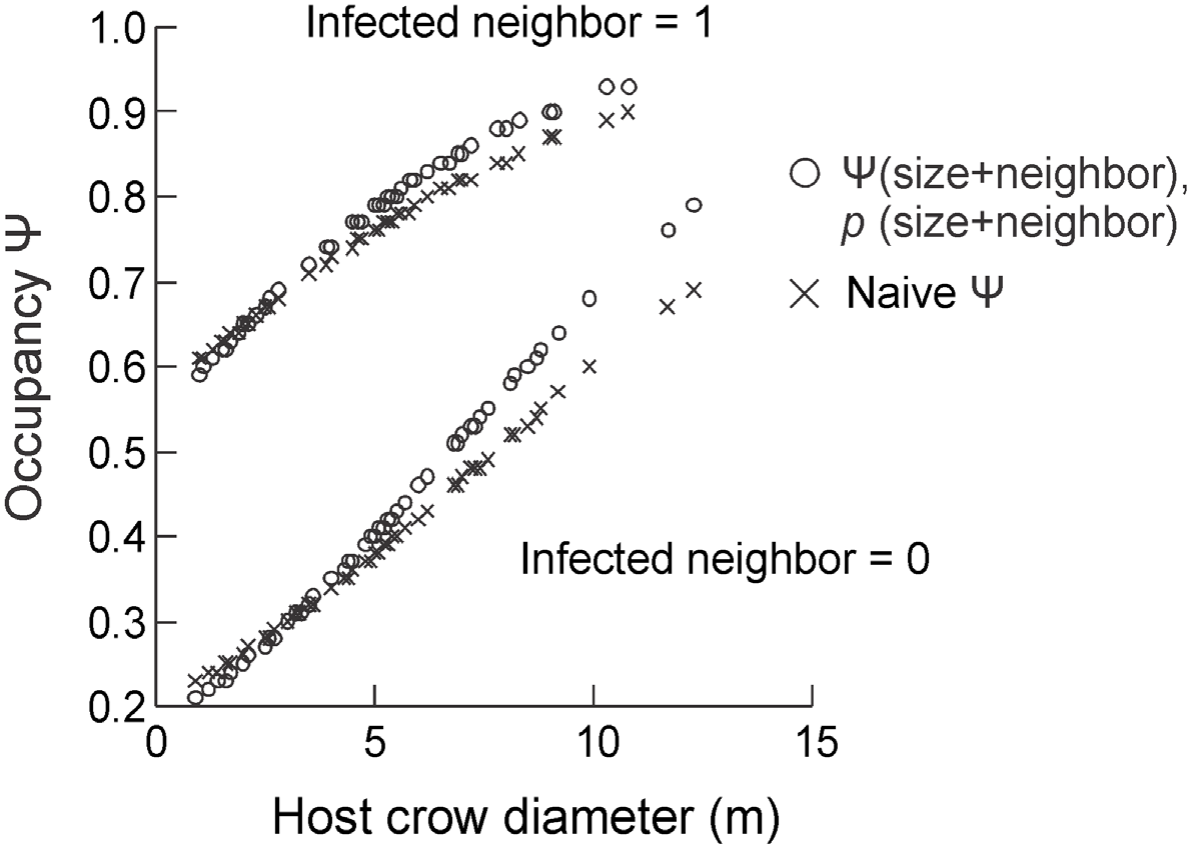
Occupancy (A) and detection probability (B) of mistletoe seeds of *Psittacanthus plagiophyllus* deposited on the host *Anacardium occidentale* according to proximity to infected hosts host size (host crown diameter). Central markers represent means, and lines represent 95% confidence intervals. Both graphs were traced with estimates from the model Ψ (size+neighborhood), p (size+neighborhood).

**Fig 3:**
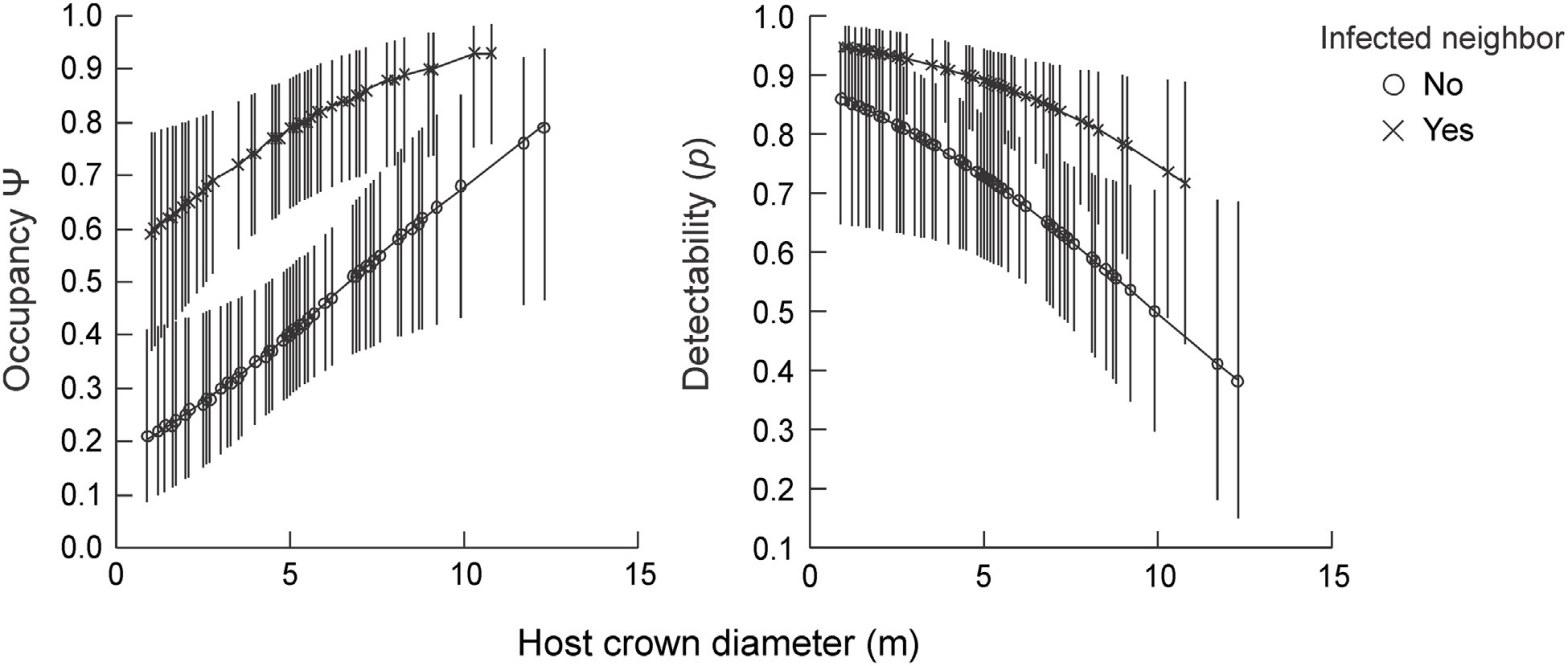
Comparison of occupancy estimates of seeds of *Psittacanthus plagiophyllus* between two models: one using naïve estimates fitted with a logistic regression (logit (p) = 0.251 + 0.18 (host crown) - 1.64(neighbor)), and other using occupancy estimates accounted for detectability (first model of Table 1).

Average crown diameter of focal host-trees was 5.3 ± 2.5 m (height: 5 ± 1.24 m). Sixty-percent (77 hosts) did not have infected neighbors, while only 9% were infected by fruiting mistletoes. Two of the thirty-six models were well supported, but the best (W = 49%) included “host size” and “presence of infected neighbor” in both terms (Table 1). As expected, occupancy was higher for larger hosts, especially for those with infected neighbors (Fig 2A). Presence of a fruiting mistletoe on the focal host, by contrast, was not an important model covariate for occupancy, nor for detectability (but note above the small proportion of hosts infected by fruiting mistletoes). Overall detectability of seeds (*p*) was high (i.e., [*p*(.)]) = 0.78; CI = 0.7–0.84). Detectability was, on average, 17% higher in focal-host trees with infected neighbors, while decreased about 23 to 50% from smallest to largest hosts (Fig 2B). Failure to detect seeds on host trees underestimated occupancy by an average of 2.5%. Occupancy was well predicted for small hosts (0.9 to 5 m of crown diameter), but was underestimated for the larger ones, especially for those without infected neighbors (~10%) (Fig 3).

**Table 1:** Summary of model selection for predicting both the occupancy and detectability of hosts (*Anacardium occidentale*) by seeds of the mistletoe *Psittacanthus plagiophyllus*.

Model	ΔAICc	W	-2l	K
Ψ (size+neighborhood), p (size+neighborhood)	320.03	49%	307.35	6
Ψ (size+neighborhood), p (size)	322.03	18%	311.55	5
Ψ (size+neighborhood), p (infection+neighborhood)	324.12	6.3%	311.44	6
Ψ (size+neighborhood), p (size)	324.31	5.7%	313.83	5
Ψ (neighborhood), p (size+neighborhood)	324.59	5%	314.11	5

Models were organized in decreasing order of importance. Only five of the 36 models and their respective resulting values are presented.

## Discussion

This is the first study to estimate occupancy of host trees by mistletoe seeds using detectability information gathered from re-surveys. We show that we failed to determine the state of hosts as occupied by mistletoe seeds in several occasions (i.e. detectability is less than one) and, therefore, we warn against the use of single visits. Although many studies collect quantitative information about host colonization by mistletoe seeds instead of occurrence data [10,15,17,30,39], we urge that detectability should not be neglected. Due to the pervasive, aggregated pattern of the seed shadow in mistletoes (where the majority of host trees receive very few seeds), not considering detectability could substantially underestimate host occupancy.

An intuitive way to compensate for potentially low detection of mistletoe seeds in metapopulation studies would be to increase the search time during single visits conducted to host trees. However, no matter how long is this, without a measure of uncertainty provided by probabilistic models, an observer could never know how precise and accurate are the estimates of occupancy [40]. Therefore, in order to increase precision and accuracy of parameter estimates in mistletoe metapopulation studies, we suggest increasing the number of independent visits conducted to the same host trees rather than expanding the time spent searching for seeds.

Our best model indicates that both occupancy and detectability varied as function of two main host covariates: size and proximity to other infected hosts. Host size affects occupancy positively probably because of the preference of bird dispersers for perching on branches of larger than average trees after feeding on mistletoe fruits [41]. On the other hand, this affects detectability negatively, as a larger host ‘dilutes’ the chance of finding seeds that are present. As predicted, host trees located close to other infected individuals had higher occupancy and detection probabilities due to the limited seed dispersal distances covered by birds after feeding on fruits, which concentrates most seeds in the neighborhood of infected hosts [18]. Finally, presence of adult mistletoes on focal trees was a poor predictor of host occupancy and detectability. This is because *Elaenia cristata* after consuming *Psittacanthus* fruits in one tree retain seeds in or on their bills until they land in another tree [17,39], therefore not increasing the chance of seed deposition on previously infected hosts.

The most important aspect to consider when dealing with detectability in metapopulation, as well as in long-term monitoring studies, is how failure to detect a species affects site occupancy estimates. In our study, this was low on average (~2.5%). However, it increases up to 10% for large hosts without infected neighbors. In an analysis of immediate relevance to the current study, Moilanen [20] showed that 10% of false-absences may influence considerably the results and conclusions of metapopulation dynamics modeled as a Markov chain process. He showed that failing to detect organisms in large patches, in contrast with a more accurate detection in smaller ones, could cause a strong overestimation of extinction rates for the former in comparison with the latter. Further, failing to detect organisms in isolated patches, followed by in subsequent seasons, can also cause overestimation of species colonization ability. In our case, if we had been interested in using a Markov chain to model metapopulation processes or mistletoe transmission from its earliest stages [13], we would have obtained a higher rate of production of new infections for the [*(t+1)*] sampling occasion because of false-absences from hosts occupied by seeds in the previous sampling time [*(t)*].

### Perspectives and improvements for future metapopulation and monitoring studies of mistletoes and other tree-dwelling plants

Suppose we were not interested in modeling mistletoe metapopulations from the beginning of the process of seed transmission, but only in successive sampling of established mistletoes on the focal host trees [e.g., 12]. Even in this case, our sampling would not be free of false-absences. In our experience based on several years of sampling mistletoes on their host trees [17,29,42], established mistletoe infections can be missed numerous times. Indeed, we also sampled mistletoe infections using presence-absence on host trees and found a detectability of 0.83 (CI = 0.75–0.89). However, we decided to remove this from this study because size of infections seems to be more important for detection than host tree covariates. In this sense, if marking mistletoes on host trees is possible, we still suggest using re-surveys to separate the process of mortality (extinction for a simple metapopulation model) and recruitment (colonization) from the detectability itself, especially for small, inconspicuous life-states of plants [23]. If appropriate to the research question, quantitative datasets could be converted to occupancy after sampling [e.g., 12].

Some studies besides the current one have already investigated empirically how host tree characteristics influence detection of mistletoe plants. For example, Geils and Mathiasen [43] first rated a stand of trees for mistletoe presence, and then conducted a detailed sampling of felled trees, showing that is difficult to rate infection intensity for both larger trees in dense coniferous forests, as well as small trees with short crowns. In contrast, Shaw *et al*. [44] used a canopy tree crane facility to evaluate the accuracy of ground-based surveys of a group of mistletoes in a sample of coniferous trees. They concluded that it is more difficult to rate trees infected with small mistletoe plants concentrated high above the ground through dense vegetation, than to assess presence of large mistletoes, or sample trees with unobstructed crowns. Many monitoring programs in the United States and Canada, such as the Forest Health Monitoring Program and the Canadian Forest Insect and Disease Survey, use presence-absence data to detect general trends of mistletoe spread, and design strategies to conduct preventive or corrective management [see references in 45]. Although these monitoring programs use careful inspection to reduce detection error, the single-survey data commonly used to perform comparisons among trees, plots or stands may be unreliable if detectability varies between sites, years or observers. Accordingly, using re-surveys to account for detectability allows the investigator to determine the degree of precision of occupancy estimates, increasing integration reliability when data has come from several sources such as plots, aerial surveys, aerial photography, road surveys, and remove sensing [46–48].

Besides mistletoes, several other studies proposed to use other tree-dwelling plants as model organisms for exploring metapopulation dynamics [49–52]. Indeed, failure to detect dispersal propagules or adult plants on host plants may not be an exclusive property of mistletoes. However, to our knowledge, only Snall et al. [53] gave a brief mention of detectability in their work (“spore capsules [of *Orthotricum obtusifolium*—an epiphytic bryophyte] are less conspicuous and hard to spot from the ground”, our square brackets), though they did not quantify the effect. We recommend using re-surveys to account for detectability in all plants for which a metapopulation approach has been applied. Because conducting repeated surveys on the same host trees maybe time-consuming, we further suggest identifying detectability problems using host or site covariates first, and then increasing survey effort for those host types (or sites) with poor detectability (e.g. larger and isolated trees in our study). Finally, we recommend that sampling is conducting independently at different time-periods for different observers; permitting the use of time-free surveys instead of the time-fixed technique that we have applied here.

## Supporting Information

S1 Dataset This file includes the raw data underlying the statistical analyses in the manuscript. (XLSX)
