# Correction: Modeling Occupancy of Hosts by Mistletoe Seeds after Accounting for Imperfect Detectability

**DOI:** 10.1371/journal.pone.0163008

**Published:** 2016-09-16

**Authors:** Rodrigo F. Fadini, Renato Cintra

The images for Figs 2 and 3 are incorrectly switched. The image that appears as Fig 2 should be Fig 3, and the image that appears for Fig 3 should be Fig 2. The figure captions appear in the correct order. Please see the corrected Figs 2 and 3 here.

**Fig 2:**
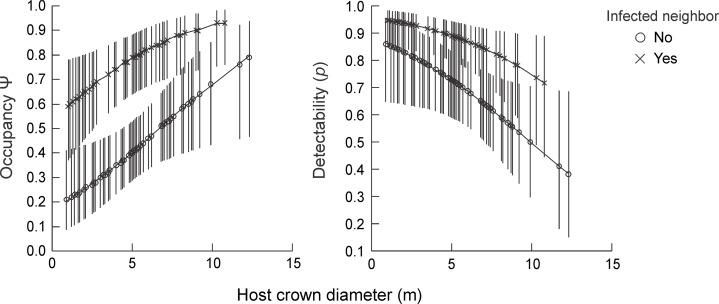
**Occupancy and detection probability of mistletoe seeds of *Psittacanthus plagiophyllus* deposited on the host *Anacardium occidentale* according to proximity to infected hosts host size (host crown diameter).** Central markers represent means, and lines represent 95% confidence intervals. Both graphs were traced with estimates from the model Ψ (size+neighborhood), p (size+neighborhood).

**Fig 3:**
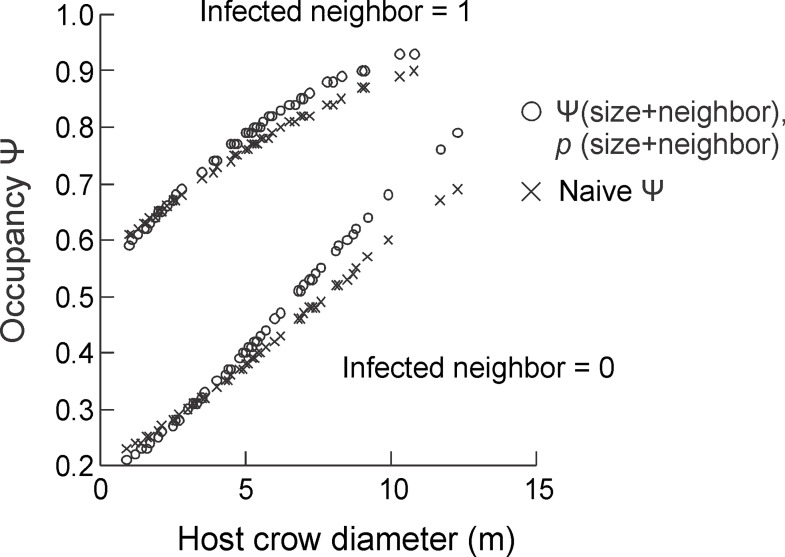
Comparison of occupancy estimates of seeds of *Psittacanthus plagiophyllus* between two models: one using naïve estimates fitted with a logistic regression (logit (p) = 0.251 + 0.18 (host crown) - 1.64(neighbor)), and other using occupancy estimates accounted for detectability (first model of Table 1).
